# Commentary: Test-Negative Design Reduces Confounding by Healthcare-Seeking Attitude in Case-Control Studies

**DOI:** 10.2188/jea.JE20180177

**Published:** 2019-08-05

**Authors:** Kotaro Ozasa, Wakaba Fukushima

**Affiliations:** 1 Department of Epidemiology, Radiation Effects Research Foundation, Hiroshima, Japan; 2 Department of Public Health, Osaka City University Graduate School of Medicine, Osaka, Japan; 3 Research Center for Infectious Disease Sciences, Osaka City University Graduate School of Medicine, Osaka, Japan

**Keywords:** hospital-based case-control study, selection bias, health conscious behavior

**Figure 1.**
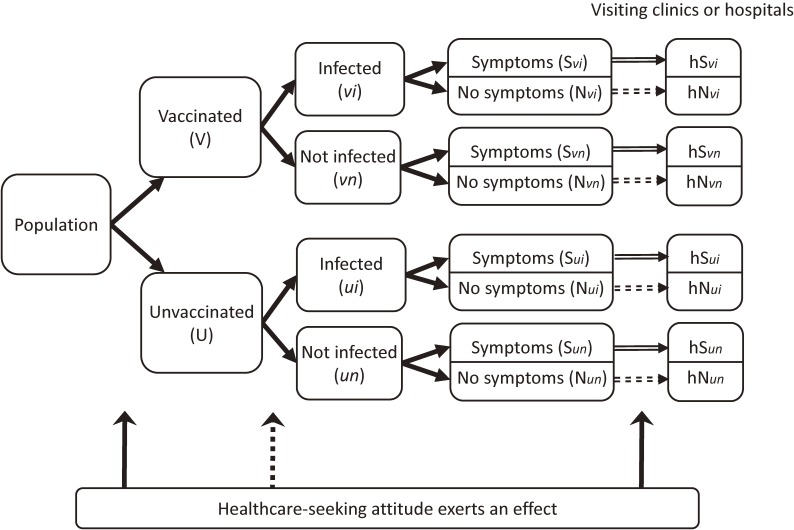
Temporal flow of vaccination, infection with target pathogens, development of symptoms, and visits to clinics or hospitals, as well as the influence of a healthcare-seeking attitude. Arrows from “Population” to “Symptoms/No symptoms” indicate choice of receiving vaccination and chance of infection with pathogens and display of symptoms. Arrows from “Symptoms/No symptoms” to boxes below “Visiting clinics or hospitals” indicate probability of the visit. Solid arrows indicate higher probability than dotted ones in general. Solid arrows from “Healthcare-seeking attitude exerts an effect” indicate definite influence on behavior of receiving vaccination and visiting clinics or hospitals, which are the major issues of this commentary. A dotted arrow indicates possible influence on chance of infection with pathogens through preventive health behaviors.

Hospital-based case-control studies employing the “test-negative design” have been used recently, with the intention of reducing confounding by healthcare-seeking attitude in the evaluation of vaccine effectiveness (VE) for some diseases with specific characteristics, such as influenza and rotavirus infection.^1^^–^^3^ Araki and colleagues investigated the effectiveness of rotavirus vaccine for children in a case-control study with this design.^1^ In this highly sophisticated study, the mechanism of reducing confounding by healthcare-seeking attitude may seem complicated, so it would be helpful for general readers for us to briefly summarize the principle as well as the benefits and cautions related to its application. Figure 1 shows the temporal flow of vaccination and course of development of the target disease; it also summarizes the evaluation of VE in observational epidemiological studies. In a target population, people are vaccinated on a voluntary basis, so that their behavior seeking vaccination might be influenced by their healthcare-seeking attitude. Later, people could be infected by the target pathogens. At this stage, opportunities for infection might be associated with preventive health behaviors that could be associated with healthcare-seeking attitudes. Subsequently, people might or might not develop the target disease, and, whether or not they develop the disease, they might or might not display symptoms of the disease. Symptomatic patients would be likely to visit clinics or hospitals. Other persons would visit clinics or hospitals for other reasons. Some patients without the target disease might display the same or similar symptoms to the target disease; for example, respiratory diseases other than influenza and gastroenteritis other than rotavirus infection. It is a key feature in a case-control study with the test-negative design that there should be such patients and they should be recruited unbiasedly regardless of diagnosis of target disease (explained later).

In a prospective study, VE for protection against clinical diseases is evaluated by the ratio of probabilities of developing disease between vaccinated and unvaccinated individuals (S*_v_**_i_*/V vs S*_ui_*/U). VE is usually expressed as (1 − probability ratio) × 100 (%), so that no effectiveness is expressed as VE = 0% (probability ratio = 1). In usual hospital-based studies, S*_v_**_i_* and S*_ui_* are approximated by hS*_v_**_i_* vs hS*_ui_*, the numbers of persons who visit clinics or hospitals. The tendency to visit a clinic or hospital (hS/S) may differ between vaccinated and unvaccinated people in accordance with their healthcare-seeking attitudes. Usually hS/S is thought to be larger in vaccinated people than in unvaccinated people, so that the probability ratio would be biased towards 1 (ie, VE would be underestimated).

In a case-control study, VE is evaluated as the ratio of odds of vaccinations among cases to the odds among controls (ie, those who did not develop the disease; note that VE = 1 − odds ratio). So, the odds ratio is (S*_v_**_i_*/S*_ui_*)/((V − S*_v_**_i_*)/(U − S*_ui_*)) in accordance with the probability ratio in a prospective study. Practical definitions of cases and controls vary by study design, especially for controls. In a hospital-based case-control study, cases are randomly selected from patients who visit clinics or hospitals and are diagnosed as being infected with the pathogens and having symptoms, so that the odds of vaccination among cases is hS*_v_**_i_*/hS*_ui_*. As hS/S is thought to be larger in vaccinated people as aforementioned, the odds tend to be larger than the typical odds of S*_v_**_i_*/S*_ui_*. If controls are randomly selected from hospital patients who neither become infected with the pathogens nor develop the symptoms of the target disease (ie, hN*_v_**_n_* and hN*_un_*), the tendency to visit a clinic or hospital (hN/N) is thought to vary according to the illness that led to seeking care. So, the direction of confounding by a healthcare-seeking attitude that is determined by hN/N and hS/S cannot be predicted.

In a case-control study with the test-negative design, controls are randomly selected from patients having symptoms that are similar to those of the target disease but who are not infected with the target pathogens (ie, hS*_v_**_n_* and hS*_un_*). Then, the odds of vaccination among controls are estimated as hS*_v_**_n_*/hS*_un_*. Here, hS/S in vaccinated patients can be assumed to be the same between symptomatic patients with and without infection (hS*_v_**_i_*/S*_v_**_i_* = hS*_v_**_n_*/S*_v_**_n_*, set to be P*_v_*) because patients do not know their infection status before visiting clinics or hospitals. The situation is the same in unvaccinated patients (hS*_ui_*/S*_ui_* = hS*_un_*/S*_un_*, set to be P*_u_*). Therefore, the odds ratio estimating the VE in the test-negative design is (hS*_v_**_i_*/hS*_ui_*)/(hS*_v_**_n_*/hS*_un_*), which can be transformed to (P*_v_*S*_v_**_i_*/P*_u_*S*_ui_*)/(P*_v_*S*_v_**_n_*/P*_u_*S*_un_*) = (S*_v_**_i_*/S*_ui_*)/(S*_v_**_n_*/S*_un_*), so that the difference in hS/S between vaccinated and unvaccinated people due to their healthcare-seeking attitude cancels.

Note that a healthcare-seeking attitude may have more complex implications in actual settings. But, the above-mentioned flow is useful for generally understanding how the test-negative design can minimize confounding by a healthcare-seeking attitude in case-control studies. During the previous decade, there has been a growing number of VE studies using the test-negative design, especially with influenza and rotavirus infections. These diseases share the characteristic that patients are likely to visit a clinic or hospital immediately after the onset of related symptoms (influenza-like illness for influenza and gastrointestinal symptoms for rotavirus infection). This characteristic enables us to apply the test-negative design with the simplified scenario of a healthcare-seeking attitude. In other words, applicability of the test-negative design depends on this characteristic (rapidly seeking medical attention).

**Figure 2.**
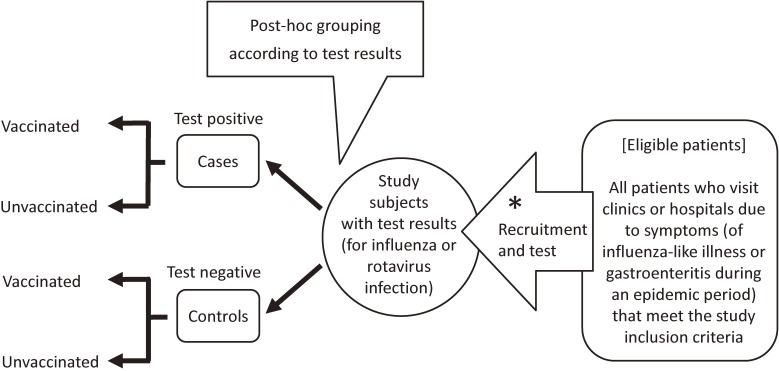
Outline of test-negative design to evaluate influenza or rotavirus vaccine effectiveness. Selection bias may occur at the procedure “recruitment and test” (^*^). So, all eligible patients (or a subset of eligible patients who are selected in a random or systematic manner) have to be recruited in the study and all study subjects (or a subset who are selected in a random or systematic manner) have to be tested. If the study subjects are limited to those who received the clinician-ordered test in a routine clinical setting, the application of the test would be related to the likelihood of having the disease under investigation (outcome) or vaccination status (exposure), resulting in biased sampling (non-representativeness) of the study subjects even if the study subjects meet the inclusion criteria. [Modified from the following source: Fukushima W, Hirota Y. Basic principles of test-negative design in evaluating influenza vaccine effectiveness. Vaccine. 2017;35:4796–4800.]

Figure 2 shows an outline of the test-negative design for VE studies of influenza and rotavirus infection, illustrating retrospective flow opposite to that of Figure 1. Patients who are eligible for the study are all who visit clinics or hospitals with influenza-like illness or gastrointestinal symptoms during an epidemic period, who meet the inclusion criteria of the study. It is essential for the test-negative design that symptoms defined as criteria for inclusion in the study should be clearly specified in advance to ensure that cases and controls have similar probabilities of visiting clinics or hospitals with regard to symptoms and healthcare-seeking attitude prior to actual diagnosis. It is also essential that, at the procedure “recruitment and test” (^*^ on Figure 2), all eligible patients (or a subset of eligible patients who are selected in a random or systematic manner) have to be recruited in the study and all study subjects (or a subset who are selected in a random or systematic manner) have to be tested; if not, sampling bias would occur. Finally, the subjects are classified into either cases or controls according to their test results for influenza or rotavirus infection. VE can be estimated as (1 − odds ratio) × 100 (%) with reduced influence of a healthcare-seeking attitude.

For many clinicians, it is likely that the test-negative design is easier to conduct than traditional case-control studies. Control selection is technically complicated in traditional case-control studies, whereas subjects who have negative test results are automatically categorized as controls in the test-negative design. This means that, in the test-negative design, investigators do not need to pay attention to whether or not the controls are selected appropriately under the specific study hypotheses. Such convenience sometimes leads to careless planning of a test-negative design without an understanding of the basic principles of epidemiology. Typical examples include influenza VE studies in Japan using information from clinician-ordered rapid diagnostic testing results in a pre-existing medical database. Since the study subjects are limited to those who received the clinician-ordered test in a routine clinical setting, application of the test would depend on the likelihood of having influenza (outcome) or influenza vaccination status (exposure), resulting in biased sampling (non-representativeness) of the study subjects even if the study subjects meet the inclusion criteria. For example, if clinicians order the diagnostic test for patients with severe influenza-like illness (ie, those with a high likelihood of having influenza), the proportion of unvaccinated persons among cases is likely to increase, leading to overestimation of VE. In order to avoid selection bias in the test-negative design, it is important to employ both “active recruitment of study subjects from the eligible patients according to pre-defined disease criteria” and “active application of the test to the study subjects” in a random or systematic manner irrespective of exposure and outcome status.^4^ A study from the United States found potential selection bias in recruitment of patients with symptoms of influenza-like illness in influenza VE studies using the test-negative design with clinician-ordered rapid diagnostic testing and indicated the importance of active unbiased recruitment of study subjects according to pre-defined symptoms and systematic testing.^5^ In Araki’s test-negative study regarding rotavirus VE, they also emphasized that the pathogenic test should be applied to all eligible patients visiting with pre-defined symptoms.^1^

Although the test-negative design is a novel modification of the traditional case-control study that reduces the biasing effect of a healthcare-seeking attitude, it is useful to understand its concept in relation to the basic principles of epidemiology. To appropriately use the test-negative design in clinical settings, guidance from epidemiologists is needed. See more detailed theoretical discussion on confounding, selection bias, and measurement error in the papers by Sullivan et al^6^ and Westreich et al.^7^
